# Central Corneal Thickness in Spectral-Domain OCT and Associations with Ocular and Systemic Parameters

**DOI:** 10.1155/2016/2596956

**Published:** 2016-06-02

**Authors:** Alexander Karl-Georg Schuster, Joachim Ernst Fischer, Urs Vossmerbaeumer

**Affiliations:** ^1^Mannheim Institute of Public Health, Social and Preventive Medicine, Medical Faculty Mannheim, Heidelberg University, Ludolf-Krehl-Straße 7-9, 68167 Mannheim, Germany; ^2^Department of Ophthalmology, Medical Faculty Mannheim, Heidelberg University, Langenbeckstraße 1, 55131 Mainz, Germany

## Abstract

*Background*. Optical coherence tomography (OCT) allows quantitative analysis of the anterior segment of the eye with a noncontact examination. The aim of this study is to analyze associations of central corneal thickness (CCT) as measured by OCT with ocular and systemic cardiovascular parameters.* Methods*. A cross-sectional study of 734 persons was performed in a working age population. Only healthy eyes were included. A comprehensive ophthalmological examination including refraction, noncontact tonometry, and imaging of the anterior segment by SD-OCT was performed. In parallel, a broad range of systemic cardiovascular parameters were measured. Associations were analyzed using a generalized estimating equations' model.* Results*. CCT measurements showed a significant association with corneal curvature and intraocular pressure: a thinner CCT was associated with a flatter cornea and with lower intraocular pressure (*p* < 0.001). Age was positively associated with CCT (*p* < 0.001); all other cardiovascular parameters were not associated.* Conclusion*. A thinner cornea is associated with a flatter surface and with lower intraocular pressure readings, while there are no independent associations with refraction and systemic cardiovascular parameters. Our findings highlight the value of SD-OCT CCT measurements as a standard tool in anterior segment analysis.

## 1. Introduction

Optical coherence tomography (OCT) enables high-resolution imaging of the anterior segment of the human eye. In a noncontact examination it is possible to analyze layers and shape of cornea in cross-sectional images. This includes the option to determine central corneal thickness.

Central corneal thickness itself affects the accuracy of intraocular pressure measurements [1]. In the last decades, correlations between central corneal thickness and intraocular pressure readings have been evaluated in many studies establishing the correlation between these two ocular parameters [2–12]. Many of these studies have used optical or ultrasonic pachymetry for corneal thickness measurements and applanation tonometry for intraocular pressure measurement. Nowadays, noncontact tonometry is widely used in clinical daily routine for screening purposes [13]. Therefore, the relationship of this measurement technique with central corneal thickness readings as determined by spectral-domain OCT (SD-OCT) imaging should be further investigated. For clinical evaluation of central corneal thickness as determined by SD-OCT, it is essential to know physiological factors that are associated with the dimensions of the tissue.

Based on this background, this study aimed to investigate the associations of distinct central corneal thickness measurements determined by SD-OCT with ocular and systemic cardiovascular parameters in a Caucasian cohort. Our hypothesis is that higher central corneal thickness may be associated with a flatter corneal curvature and with higher intraocular pressure, while systemic cardiovascular parameters are not associated.

## 2. Materials and Methods

The study was designed as a cross-sectional, observational study conducted within the scope of a health promotion project conducted within a major industry company. The study population consists of working age subjects (age range from 17 to 64 years) of the MIPH Eye & Health Study. Exclusion criteria were any manifest eye disease, either self-reported or detected on fundus photographs, and insufficient OCT scan quality of the anterior segment OCT. 1460 healthy eyes of 734 subjects (730 right eyes, 730 left eyes; 514 men, 220 women) were included in the analysis. The characteristics of the sample are given in Table 1.

The study was conducted according to the tenets of the Declaration of Helsinki and fully approved by both the ethics approval committee of the University of Heidelberg (institutional review board), the company's advisory board, and the board of employees. Written informed consent as to the scientific objectives of the study was obtained from each participant.

Blood pressure was recorded from the dominant arm in the seated position after a standardized 20 min rest period by sphygmomanometry. Body weight and height were measured and body mass index was calculated. Blood samples were drawn to determine triglycerides (TRI), low density lipoproteins (LDL), high density lipoproteins (HDL), and glycosylated hemoglobin (HbA1c) using routine laboratory analyzers.

The cornea was imaged with the anterior segment mode of the 3D OCT-2000 (Topcon Corp., Tokyo, Japan). Automated calculation of corneal curvature and central corneal thickness (CCT) with the integrated software was performed and all OCT images were checked for correct identification of the corneal surface. Quality of OCT scans were graded in a four categories: “high,” “medium,” “acceptable,” and “insufficient.” Mean curvature of the corneal radius was computed. Automated objective refractometry was obtained using a KR-8900 (Topcon Corp., Tokyo, Japan) and noncontact tonometry was performed (CT-80 Tonometer, Topcon Corp., Tokyo, Japan). Three averaged measurements were obtained per eye.

Fundus photographs of the macula and optic nerve head were obtained from all participants and images were evaluated by two independent ophthalmologists. Eyes with previous intraocular or refractive surgery were excluded from the study, as were eyes with known and confirmed ocular disease. Subjects wearing contact lenses were excluded as this might affect corneal thickness. All other eyes were included.

Raw data results were processed by statistical analysis software (SPSS 21.0, Chicago, IL) and plots were performed with STATA software (version 13.1 SE, College Station, TX: StataCorp LP). Associations with central corneal thickness were analyzed by multivariable linear regression algorithms with parameter estimation performed with generalized estimation equations. This statistical model takes the relationship between the right and left eye into account by linking the individual pair of eyes. As dependent variable central corneal thickness was included in the statistical model, mean corneal radius, intraocular pressure, refraction, age, gender, body mass index, mean arterial blood pressure, HbA1c, HDL, LDL, and triglyceride values were evaluated as associated factors.

Association analysis between central corneal thickness and intraocular pressure, respectively, means corneal curvature was performed with univariate linear regression as well to determine comparable parameter estimates with literature reports. In addition, a reverse analysis with intraocular pressure as dependent and central corneal thickness as explanatory variable was computed. Spearman correlation coefficient was computed to determine the relationship between central corneal thickness readings and OCT image quality.

A *p* value of 0.05 was regarded as statistically significant. Due to multiple testing, we corrected this parameter to 0.004 using the Bonferroni method. For further interpretation of the results, all *p* values above 0.001 are exactly reported.

## 3. Results

**Figure 1:**
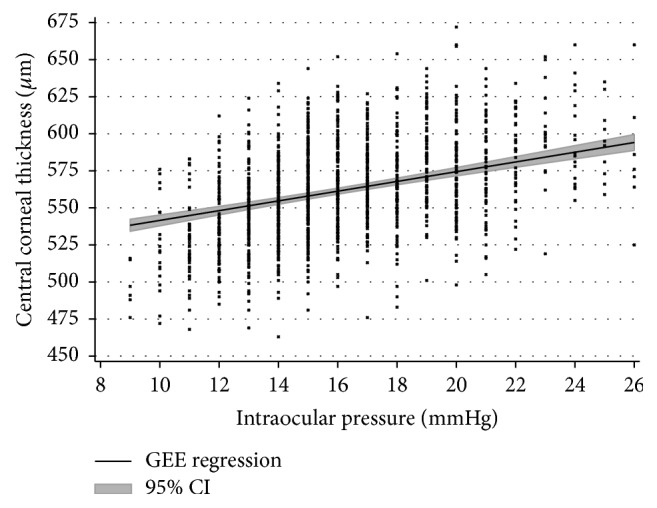
Association of central corneal thickness (CCT) as determined with SD-OCT and intraocular pressure (IOP). The correlation coefficient is *r* = 0.45 (*p* < 0.001) and the regression equation is CCT (in *µ*m) = 509 + [3.29 *∗* IOP (in mmHg)].

**Figure 2:**
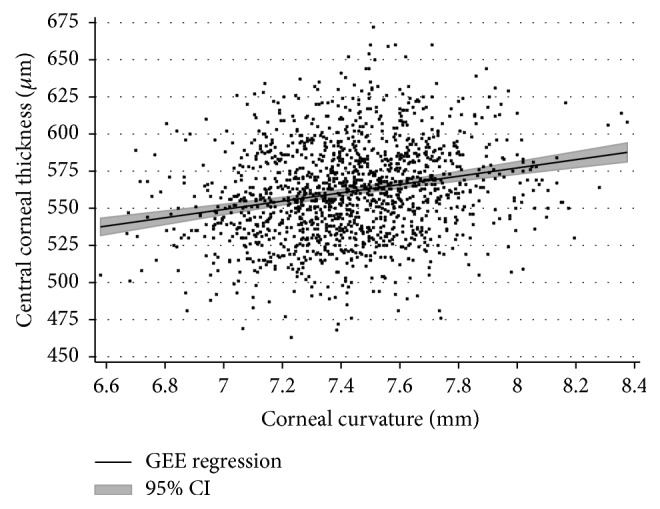
Relationship of central corneal thickness as determined with SD-OCT and corneal curvature: an increase of central corneal thickness (CCT) is associated with a flatter corneal curvature (CC). The correlation coefficient is *r* = 0.20 (*p* < 0.001) and the regression equation is CCT (in *µ*m) = 354 + [27.9 *∗* CC (in mm)].

Considering all eyes together, mean CCT was 561.1 ± 32.3 *µ*m (mean ± standard deviation; right eyes: 560.1 ± 31.8 *µ*m; and left eyes: 562.2 ± 32.8 *µ*m). Association analysis of CCT measurements found a statistically significant relationship between corneal curvature and intraocular pressure: CCT was found to be negatively associated with intraocular pressure readings (*p* < 0.001; Figure 1) and positively associated with a flatter corneal curvature (*p* < 0.001; Figure 2). Reversely, univariable analysis between intraocular pressure as dependent variable and central corneal thickness as explanatory variable revealed that an increase of 22 *µ*m in central corneal thickness is linked to an increased measurement of intraocular pressure of 1 mmHg.

In the multivariable model, an increase of 10 *µ*m in central corneal thickness was associated with an increase of 3 mmHg in intraocular pressure reading. Refraction of the eye did not show a significant association with CCT (Table 2). Regarding anthropometric characteristics of the study sample, age was independently associated with CCT: older subjects had a higher central corneal thickness (Table 2). Central corneal thickness values from OCT measurements increase by 0.34 *µ*m per year of age.

Regarding cardiovascular parameters, none of the investigated parameters (gender and BMI and mean arterial blood pressure and biochemical parameters: HbA1c and HDL and LDL and triglycerides) was significantly associated with CCT after Bonferroni correction for multiple testing (*p* < 0.004) (Table 2).

A weak association between lower scan quality and thinner central corneal thickness measurement was determined (Spearman correlation coefficient *ρ* = −0.09, *p* < 0.001). Nevertheless, upon incorporating this parameter as sensitivity analysis in the multivariable model, our findings remained unaltered.

## 4. Discussion

Our study in a Caucasian cohort demonstrates that central corneal thickness readings as measured with the Topcon 3D OCT-2000 are independently associated with corneal curvature and with intraocular pressure readings. Previously published studies have yielded congruent findings using optical or ultrasound pachymetry and association with intraocular pressure [2–4, 7, 8, 10]. However, we used SD-OCT for determining central corneal thickness which makes a technical difference to other studies where analyses were based on ultrasound pachymetry or Scheimpflug imaging. SD-OCT accurately measures central corneal thickness as reported by prior publications within the physical limitations of the method itself as determined by wavelength, optical system, and sensor characteristics [14]. Furthermore, our finding of a relationship of corneal curvature with central corneal thickness as measured by OCT is new. Nevertheless, there are several studies reporting such a relationship using other methods [15–18]. Also, our study may contribute to deepening the understanding of associations between CCT readings obtained from OCT and intraocular pressure readings obtained from noncontact tonometry and add to knowledge being generated with different methods [12]. In the range between 11 and 21 mmHg intraocular pressure and 500 to 625 *µ*m central corneal thickness, we found an approximately linear relationship between these two parameters, but this relationship cannot be extrapolated to higher or lower values as measurements were not sufficiently available there. With an increase of 22 *µ*m in CCT, intraocular pressure (as dependent variable) increased by 1 mmHg, and this magnitude is similar to that reported in literature [12].

There are few studies about systemic influencing factors on central corneal thickness. Elflein et al. reported associations of central corneal thickness with male gender, higher body height, and body mass index in a German population [19]. The differences with our study might be explained by the underlying study population: we included far more male than female participants, a different age span (17 to 64 years versus 35 to 74 years by Elflein et al.), and a different mean body mass index (22.9 versus 27.2), while both studies included mainly Caucasians. In a similar age span to Elflein et al., Nangia and coworkers found associations of central corneal thickness with male gender, younger age, and higher body mass index in an Indian population [8].

In Chinese people, central corneal thickness is associated with male gender and urban region, while it is not associated with age, body mass index, and body height [7]. A study in a Japanese population found similar findings for Asian people reporting an association of central corneal thickness with male gender but none for age, body weight, and body height [11]. The ambiguities to our study results may arise from the use of different CCT assessment methods and variations in age, body measurements, and genetic background of the study populations.

In our study population consisting of a cohort of Caucasians with an age range from 17 to 64 years, central corneal thickness was associated with age: each decade of age went along with an increase of 3.4 *µ*m in central corneal thickness over the entire age span. Upon comparing our findings to those published by other groups, it must be considered that our age range corresponds to the working age and that we have only included healthy eyes with the purpose of reporting physiological conditions. This means that our study group starts at a juvenile age and terminates at retirement, whereas many other studies have appeared with a much older starting age reaching into senility. There are some studies in European cohorts with different age ranges that did not find an association between central corneal thickness and age [9, 10, 20]. In contrast, we found an association with age: central corneal thickness increased slightly up to the age of 64 years. In other ethnicities and especially in persons aged 70 years and older, Chua et al. reported a decrease of central corneal thickness with age [21]; similar results were reported by other research groups [22–24]. This indicates that central corneal thickness may gradually increase in the decades covered by our cohort, while over 70 years a decrease may be observed.

In contrast to earlier studies, gender and body mass index were not related to central corneal thickness, nor was any other cardiovascular parameter. Interestingly, mean arterial blood pressure showed some association with CCT. However, this association was only small and failed to reach the level of significance after Bonferroni correction. Sensitivity analysis revealed that systolic blood pressure is rather associated with central corneal thickness compared to diastolic blood pressure. Based on these negative findings for associations between central corneal thickness and cardiovascular parameters, the underlying hypothesis seems to be true that central corneal thickness itself is independent of cardiovascular changes.

Our study has several limitations: first, we did not examine axial length and consequently could not control for it as influencing factor, as previously reported by Nangia et al. [8]. As refractive power of the cornea is known to be associated with central corneal thickness [8], we included both corneal curvature and overall manifest refraction into our analysis model: we found an association of central corneal thickness with corneal curvature, but not with refraction after having corrected for corneal curvature. Though not expected, this finding is similar to the study report by Zhang et al. [7], who also did not find an association of central corneal thickness with refraction. In addition, due to the fact that our study cohort is a cross section of a working population, this may not strictly reflect the entire Caucasian population. Nevertheless, we examined a cohort of over 700 subjects with a broad range of age (from 17 to 64 years) and refraction (from −11.75 D to +7.625 D) which may come close to this criterion. OCT scan quality is a critical issue for further data processing [25, 26]. We found that there is a correlation between lower OCT scan quality and thinner central corneal thickness measurements; nevertheless, the findings for associated factors did not alter upon including the OCT scan quality parameter.

In conclusion, our study evaluated associations of central corneal thickness readings determined by SD-OCT in healthy eyes. As our study was explicitly focused on healthy eyes, this approach may be worthwhile for defining norm values for this specific technology. Also the method determining intraocular pressure differs from other studies. Analysis confirmed intraocular pressure and corneal curvature as ocular factors associated with central corneal thickness. Regarding cardiovascular factors, central corneal thickness was not associated with any examined parameters except age. These data suggest that clinical evaluation of central corneal thickness as determined by SD-OCT should also be performed with respect to individual corneal curvature and intraocular pressure interpretation merits the knowledge of central corneal thickness.

## Figures and Tables

**Table 1:** Characteristics of the study sample (*n* = 734 participants). OD: right eyes, OS: left eyes; myopic, emmetropic, and hyperopic eyes classified according to spherical equivalent measurements.

	Mean	Standard deviation	Minimum	Maximum
Age (years)	40.4	11.2	17	64
Body mass index (kg/m^2^)	22.9	4.0	15.2	49.3
Mean arterial pressure (mmHg)	98	11	70	148
HbA1c (%)	5.62	0.40	4.60	9.60
High density lipoproteins (mg/dL)	59	13	28	113
Low density lipoproteins (mg/dL)	126	32	54	258
Triglycerides (mg/dL)	126	78	30	741
Manifest refraction (diopters):				
Sphere OD	−1.61	2.70	−9.75	6.50
Cylinder OD	−0.68	0.74	−6.00	0.00
Sphere OS	−1.59	2.82	−11.00	8.50
Cylinder OS	−0.73	0.81	−6.25	0.00
Tonometry (mmHg):				
OD	16.01	3.23	9	26
OS	16.21	3.22	9	26
Central corneal thickness (*μ*m)				
OD	560.1	31.9	468	672
OS	562.2	32.9	463	660
Anterior corneal curvature (mean radius in mm)				
OD	7.43	0.26	6.68	8.31
OS	7.43	0.28	6.58	8.38

Manifest refraction: grouped	Frequency	Percentage		

Right eyes:				
Myopic eyes (<−0.5 D)	*n* = 430	59%		
Emmetropic eyes	*n* = 232	32%		
Hyperopic eyes (>0.5 D)	*n* = 68	9%		
Left eyes:				
Myopic eyes (<−0.5 D)	*n* = 441	60%		
Emmetropic eyes	*n* = 218	30%		
Hyperopic eyes (>0.5 D)	*n* = 71	10%		

**Table 2:** Association analysis of SD-OCT based central corneal thickness on ocular and systemic parameters using a generalized estimating equations model. Regression coefficient Beta (Reg *β*), 95% confidential interval (95% CI), and *p* value.

Central corneal thickness	Associations		
Multivariable linear regression	Reg *β*	95% CI	*p* value
Mean corneal radius [mm]	25.19	[19.25; 31.12]	0.000
Intraocular pressure [mmHg]	3.33	[2.75; 3.92]	0.000
Refraction [SE in diopters]	0.31	[−0.28; 0.91]	0.30

Age [years]	0.34	[0.15; 0.53]	0.001
Gender [female]	1.95	[−3.52; 7.41]	0.49
Body mass index [kg/m^2^]	0.23	[−0.42; 0.87]	0.49
Mean blood pressure [mmHg]	−0.31	[−0.52; −0.09]	0.005
HbA1c [in %]	3.24	[−3.77; 10.25]	0.37
High density lipoproteins [mg/dL]	−0.23	[−0.42; −0.05]	0.012
Low density lipoproteins [mg/dL]	−0.06	[−0.13; 0.02]	0.13
Triglycerides [mg/dL]	0.01	[−0.02; 0.04]	0.44
